# Current trends on the application of artificial intelligence in medical sciences

**DOI:** 10.6026/973206300181050

**Published:** 2022-11-30

**Authors:** Aisha Mousa Mashraqi, Budoor Allehyani

**Affiliations:** 1 Department of Computer Science, College of Computer Science and Information Systems, Najran University, UAE; 2 Department of Information System, College of Computers and Information Systems, Umm Al-Qura University, UAE

**Keywords:** Machine Learning, Deep Learning, Disease Diagnosis, Medical Imaging, Drug repurposing

## Abstract

Artificial Intelligence (AI) is expanding with colossal applications in various sectors. In the healthcare sector, it is booming to make life simpler with utmost accuracy by predicting, diagnosing and up to care with the help of Machine Learning (ML) and Deep Learning (DL) applications. Modern computer algorithms have attained accuracy levels comparable to those of human specialists in medical sciences, although computers often do jobs more quickly than people do. It is also expected that there will not be a mandate for humans to be present for the jobs that machines can do, and it is gaining the highest peak because of good trained artificial models in the medical field. ML enhances the therapeutic process and improves health by encouraging more patient participation. ML may get more accurate patient data when used with the Internet of Medical Things (IoMT) and automate message notifications that prompt patients to respond at certain times. The motivation behind this article is to make a comprehensive review of the on-going implementation of ML in medical science, what challenges it is facing now, and how it can be simplified for future researchers to contribute better to medical sciences while applying it to the practitioners' jobs easier. In this review, we have extensively mined the data and brought up systematised applications of AI in healthcare, what challenges have been faced by the experts, and what ethical responsibilities are liable to them while taking the data. We also tabulated which algorithms will be helpful for what kind of data and disease conditions will be useful for future researchers and developers. This article will provide a better insight into AI and ML for the beginner to the advanced developer and researcher to understand the concepts from the basics.

## Background:

In 1959, Arthur Samuel, a renowned computer scientist, defined ML as the "computer's ability to learn without being explicitly programmed". As it completes task T with a performance measure P, a computer program learns from experience E. The task performance improves with experience [1, 2]. For simplicity, we can take an example of the checkers game, where the program learns good and bad positions to win a game through experience gained by playing against itself. ML is a sub-domain of AI, which aims to build machines as intelligent as a human brain. Algorithms are collections of mathematical operations that define the connections between variables and are the foundation of ML techniques. The ML dataset is divided into training and testing data. The division is done in a ratio of 80:20 (training: testing) and runs simultaneously [3]. To note, the training data is used for evaluation purposes, and testing data is used for validation purposes. ML encompasses various learning methods broadly classified into supervised and unsupervised learning.

## Supervised Learning:

Supervised ML (supervised ML) refers to methods where a model is trained on various inputs or features connected to a known outcome. For example, in medicine, a model can be trained to a person's lifestyle features (like smoking, eating habits, exercise) to a particular onset of disease, say suppose, diabetes [4]. After dividing the data and training the algorithm, the model is now prepared to predict the early onset of diabetes. The predictions made by the trained model can be discrete (0 or 1, positive or negative) or continuous (values can be in decimals). The model which gives discrete predictions is called classification, and the one which gives continuous prediction values is called regression [4, 5]. Classification-related issues are relatively common in medicine. In many clinical circumstances, a doctor's diagnosis of a patient involves classifying the illness based on a particular collection of symptoms. Forecasting numerical results, such as the projected duration of hospital stay, given a specific collection of data, such as vital signs, medical history, and weight, is a common focus of regression concerns.

## Unsupervised Learning:

Unsupervised learning has no predetermined results (labels). Without human input, computers search for patterns in unsupervised learning, which is also exploratory and used to locate murky groupings or patterns in datasets. These techniques are usually called dimension reduction, including factor analysis and principal component analysis. The most common and well-known techniques are K-means clustering and DL, while DL may also be used in a supervised environment [4]. These algorithms also do association tasks that resemble clustering. These methods are unsupervised since no human input is provided on which set of attributes the clusters should be focused on. The increasing adoption of ML in many industries, including healthcare, has been made possible by advancements in data technologies, including storage capacity, computing power, and data transport speeds [6]. The difficulties of delivering appropriate healthcare to everyone have been underlined by contemporary medical developments, which emphasise the necessity for a "precision medicine" or personalised medicine approach to healthcare. Personalised medicine seeks to find, anticipate, and analyse diagnostic choices using enormous amounts of healthcare data so that physicians can subsequently apply them to each patient [7]. A few examples of the information that might be used include data on a person's genes or family history, medical imaging data, medication combinations, patient health outcomes at the community level, and natural language processing of prior medical records. The development of alternative staffing models and IP capitalisation, the provision of competent healthcare, the reduction of administrative and supply costs, and the vital roles they play in patient care, billing, and medical records are all made possible by technology today. One such subject that is gradually gaining traction in the healthcare sector is ML. To detect malignant tumours in mammograms, Google recently built a machine-learning system. Stanford University researchers now use DL to detect skin cancer [8, 9]. ML is already helping in various healthcare scenarios. Numerous more applications of ML in healthcare include providing exact resource allocation, timely risk assessments, and analysis of hundreds of distinct data points to suggest outcomes. A few applications are listed in Figure 1(see PDF).

The term "medical imaging" refers to a range of techniques used to create pictures of the inside of the human body for use in the diagnosis, analysis, and medical intervention. This is one of the three main applications of ML. The need for exploratory surgery, a discredited clinical standard, is reduced or eliminated. Since accessing any area of the human body by surgery raises the risk of infections, strokes, and other complications, medical imaging is currently the advised way for early diagnosis in the clinical environment [10, 11]. Medical Imaging- Lesion and automated computer detection are discovered in body scans such as mammography and brain scans. On the other hand, scanning these documents and incorporating the generated pictures into a database are two methods used in medical records' natural language processing (NLP) to extract meaningful information from the free text [12, 14]. They include image processing as well as recognising ideas and phrases. In handwritten medical records, complaints from patients, observations made by the doctor, and family history are all recorded. This medical information may be annotated. However, due to the doctor's shoddy writing, it could be challenging to identify this information correctly. Although there is still a risk of missing data, analysing language in pre-structured forms and papers is simpler. Cases of NLP: A recent study published in Nature Communications employed machine learning approaches to automatically gather GWAS data from open-access journals and extract GWAS associations into a database to assist curators [15, 16]. Despite mixed findings (60-80% recall and 78-94% precision), this is just one of the various ways NLP is used to advance medical research. A different study used NLP to categorise risk-stratify individuals with cirrhosis. This research could identify cirrhosis patients using electronic health data, ICD-9 code combinations, and radiological imaging with a 95.71 sensitivity and 93.88 specificities. Jenni Sidey-Gibbons (2019) observed that medical researchers and physicians are very interested in ML methods in the article "ML in medicine" [4]. The researchers showed how to accurately categorise tumour samples as benign or malignant using three widely used algorithms: a regularised general linear model, artificial neural networks and support vector machines (SVM) [4]. The algorithm can predict outcomes when used with new data after it has successfully been trained. The assessment and validation samples were chosen randomly from the publicly accessible breast mass sample dataset. Before they were used to forecast the diagnostic result in the validation dataset, the algorithms were trained on data from the evaluation sample. The trained algorithms had high specificity (.99), sensitivity (.99), and accuracy (.94–.96) when classifying cell nuclei (.85 - .94). The SVM technique produced the highest accuracy (.96) and area under the curve (.97). When algorithms were grouped into a voting ensemble, prediction performance somewhat improved (accuracy=.97, sensitivity =.99, specificity =.95) [4, 17]. Figure 2 (see PDF) shows how AI can be implemented in cancer diagnosis with the highest accuracy.

## Application areas of AI in Healthcare:

Right now, we are in the era of AI and realise the enormous potential and advances that ML has brought to the healthcare profession. AI is not new to the medical field, and the dramatic increase in the use of AI in healthcare over the last ten years can be seen in any medical hospital cum institution where research and treatment are being provided [18, 19]. ML outperforms humans in illness diagnosis in terms of speed. Malignant tumour detection algorithms are becoming more accurate than human radiologists. In order to enhance the standard of care, neural networks may now analyse outside data on a patient's status, including their X-rays, CT scans, other diagnostics, and screenings. DL makes it possible to distinguish between cancer cells and healthy ones. The model is given several images of healthy and cancerous cells so that it may "memorise" how they appear by identifying patterns. However, the complete replacement of people in medicine is still a long way off. Here, we cover the potential applications of AI in healthcare and the barriers to broader use. Many tools have been used to screen the model, but they are using various algorithms that can also be implemented in ML for better training and testing and can be converted to the various healthcare sectors [20, 21, 23, 24, 25, 26, 27, 28, 29, 30, 31]. 

## Potential of ML in Healthcare:

One of the most popular branches of AI is ML. It is based on training the model with provided data and predicting its correctness by testing it with anonymous data. Deloitte found that 63% of the 1,100 US businesses utilising AI concentrated on ML [28, 30, 32]. The industry's organizational side may benefit from the application of ML. A typical nurse in the US devotes 25% of her working hours to administrative and regulatory tasks. Technology may easily replace these tedious operations, including claims processing, revenue cycle management, clinical documentation, and records management. More than three hundred healthcare executives and clinical professionals said there is an issue with patient involvement in a different study conducted by the Harvard Business Review. Better patient health outcomes may result from more patient participation. ML may advance by providing automatic message warnings and pertinent, tailored material that prompts actions at crucial junctures. In general, there are several ways that ML may individualise and enhance therapeutic procedures [33, 34].

## Applications of ML in Healthcare and Medicine with High Precision:

The bot system is one of the most acceptable ML applications for healthcare, making the treatment process much more convenient. A virtual nurse may serve as a voice-activated healthcare assistant who informs patients on various diseases, physical conditions, and medications. 

An AI assistant might be helpful when a patient requires immediate assistance but finds it difficult to reach the doctor. Data engineers create solutions for all medical operations, including diagnosing, treating, and preventing disease. In Figure 3(see PDF) a few noteworthy applications are shown. In addition to the predominance of chatbots, we may focus mainly on the use of ML in Healthcare algorithms in:

 • Pathology

 • Oncology

 • Rare diseases

Artificial ML methods are used in the healthcare industry, mainly in neural networks. Convolutional neural networks, for instance, can identify, detect, and recognize images. To "memorise" and forecast patterns in the appearances of normal cells vs pathological cells, these are a complex system of artificial neuron layers connected and pre-trained on a dataset of normal and abnormal cells (e.g., cancer cells) photos. Implementing patient diagnostic mobile apps based on CNN may be advantageous for medical facilities and academic institutions [35, 36]. Table 1 (see PDF) displays a few uses of AI in healthcare.

An automated model can analyse information twice as quickly as human sight without needing a microscope. ML's capacity to analyse organic fluids of patients (tissues, blood and urine) may also be helpful to a pathologist's job since it can do so more quickly and accurately [37, 38]. However, we must acknowledge that there is still a dearth of knowledge on the causes of rare illnesses and how these diseases are connected to specific traits of those afflicted. As long as they need DL, the most challenging field of ML, these medical specialities provide frequent challenges for data scientists [39, 41]. However, the application of ML in healthcare may be made in various methods. 

## World's Top ML Applications in Healthcare:

The healthcare sector is moving towards AI-based innovation, which makes AI an irreplaceable element as it eases professional medical jobs with accuracy. While Stanford University researchers can identify skin cancer using DL, Google has already produced an algorithm that successfully diagnoses cancer in mammograms [42, 43]. As a result, AI can process numerous data points, accurately forecast risks and outcomes, and perform various other tasks.

## Identification of the illness and diagnosis:

ML has improved its diagnostic capabilities and is now one of the most productive fields. The early stages of many cancer and genetic illnesses are difficult to identify, but ML could manage many of them, and a prominent example is Internationa Business Machine (IBM) Watson Genomics [44, 45]. This research assists in establishing a speedy diagnosis by combining cognitive computing with genome-based tumour sequencing. A realistic approach to using AI to enhance diagnosis and treatment in conventional hospitals is being developed by P1vital under the name PReDicT (Predicting Response to Depression Treatment) [46].

## Better health records:

Even with all these technological advancements, maintaining health data remains challenging. Support vector machines and OCR recognition algorithms might be used to categories records. The best examples are Math Works' ML handwriting recognition system and Google's Cloud Vision API [47, 48].

## Diagnosis of diabetes:

One of the most prevalent and dangerous illnesses is diabetes. It not only harms a person's health in and of itself, but it also triggers several other severe disorders. Diabetes primarily affects the heart, nerves, kidneys, and ML, potentially saving lives by predicting and suggesting better options. A system that predicts diabetes may be constructed using classification algorithms like Naive Bayes, KNN, and Decision Tree. When it comes to performance and calculation time, Naive Bayes is the most effective [49].

## Detection of liver disease:

The liver is essential for metabolism. It is susceptible to cirrhosis, liver cancer, and chronic hepatitis. Although it is challenging to forecast liver illness using vast medical data accurately, there have already been some notable changes in this field. These distinctions are made by classification and clustering algorithms used in ML. It is possible to utilize the Liver Disorders Dataset or the Indian Liver Patient Dataset [50].

## Finding the best cure:

ML is another everyday use in the first stages of medication research for patients. Microsoft's AI-based technologies are used in Project Hanover, which aims to find personalized drug combinations to treat acute myeloid leukaemia [51].

## Image analysis for diagnoses:

With its InnerEye initiative, Microsoft seeks to revolutionise healthcare data analysis. This company analyses medical photos using computer vision to diagnose patients. As technology progresses, InnerEye makes even greater waves in healthcare analytics software [52, 53].

## Personalising treatment:

One notable example in this regard is IBM Watson Oncology, which offers a variety of treatment approaches after first analysing a patient's medical history. Developing individualised treatment regimens will improve as sophisticated biosensors become more widely available and provide algorithms with more data [54].

## Adjusting behaviour:

A B2B2C-based startup called Somatix has an app that may provide advice on what you can do daily to avoid cancer. This programme tracks our daily unconscious behaviours and warns us of those that may be risky in the long run.

## Medical research and clinical trial improvement:

Clinical trials may take years to complete and need substantial expenses. Based on elements like a person's history of medical visits or medication, ML can provide predictive analytics to find the best candidates for clinical trials [ 55]. Additionally, the system will reduce data inaccuracies and could recommend the ideal sample sizes for testing.

## Using data from Medical Crowdsourcing:

Scientists have access to many data that patients have made available. A need for the future is the availability of open sources for ML applications in medicine. The value of data analytics in healthcare has already been shown by collaboration between Medtronic and IBM, enabling the capacity to understand, gathers, and make insulin information accessible in real-time. The opportunities will grow as the Internet of Things develops. Public data will also facilitate diagnosis and the issue of medicinal prescriptions [56].

## Control on Epidemic:

Talking about data analytics in 2022, professionals will have access to data from video streams, news websites, social media trends, and satellites. All the information might be processed by neural networks, which could then draw inferences about global pandemic outbreaks. Before they might do significant harm, dangerous illnesses may be managed with precise methods. It is crucial in third-world nations where there are no sophisticated medical systems. ProMED-mail, a reporting tool that operates online and keeps track of epidemic reports from around the world, will likely serve as the most outstanding example of this field [57]. Today, AI is widely used in food safety to assist in avoiding pandemic illness on farms.

## AI in Surgery:

Given the increasing need for healthcare services globally, this is also one of the most well-liked applications for ML. The following types of robotic surgery may be made:

Suturing that is automatic.

Modeling of the surgical workflow.

Material advancements for robotic surgery.

Surgical skill assessment.

Robots can now help doctors operate surgical instruments, even if it is too soon to speak about them doing all procedures. It is anticipated to develop into a specific industry with roughly $39 billion in the capital during the next five years. The robot will use its robotic hands to get the necessary medical supplies for the doctor during surgery. This procedure might save the patient's time in the operating room by around 20% while reducing surgical problems by 50%. As they gather information on each AI Surgery, ML algorithms for healthcare data analytics also evaluate and define the new potential for future procedures [58]. There is a widespread fear that AI will eliminate jobs. However, it is anticipated that automation brought on by AI would result in more employment being created than lost. Furthermore, 2 million more employees are expected to be created by AI by 2025. This is because AI and ML can only automate everyday operations, some of which are pretty complex. Thus, this allows human experts to take over more sophisticated tasks. Further, how AI can be used in different healthcare points is provided in Table 2(see PDF).

## Ethical specs of ML in Medical Sciences:

The last decade has seen tremendous increases in the application of computational techniques, particularly ML and AI, in medical sciences. As the healthcare system is transitioning from traditional to digital more data is expected to be generated, which will further pave the way for the use of ML and similar techniques for purposes ranging from diagnosis, prognosis, clinical support, drug designing and many others [59]. Promising studies and research are being carried out further to explore the possible applications of AI in healthcare, but one aspect that is being overlooked in all this is the ethical aspect of using ML techniques. It can be verified through a simple search on PubMed that when keywords such as ML and AI combined with medical science are used, we see an increasing trend in the number of publications. More interestingly, a sharp increase has been observed in recent years, but when these keywords are combined with ethics, we do not see such a sharp increase [59, 60]. If we go back to 2013, we struggle to find publications describing ML's ethical side in medical sciences. Though few countries have created guidelines on ethical responsibility regarding privacy, many other contexts are shown in Figure 4 (see PDF).

## Futuristic Gaps where AI/ML can be applied in Medical Sciences:

The ability of ML to handle healthcare data in novel ways, improve patient care, and streamline administrative procedures can upend the medical sector. Terabytes of medical records that formerly required human interpretation may be utilised as data input for ML projects in the medical sector. Without additional programming, algorithms are used in the machine learning branch of AI to learn from data. Various healthcare data types may be processed using AI. For organised data, common AI approaches include deep learning, classical support vector machines, and neural networks. For unstructured data, standard AI techniques include natural language processing. Due to its effectiveness in clinical trials, instrument development, and other areas, ML has demonstrated its value in the healthcare industry. When ML and healthcare are merged, a variety of data is produced that can help with improved patient analysis, prevention, and treatment [61]. Microsoft's Project InnerEye ML methodologies were employed in a test case to distinguish and sort tumours using 3D radiology outputs [53]. It can aid in accurate navigation, tumour-contouring for radiation planning, and operation planning. ML algorithms are being added to MRI and other sophisticated imaging systems, which are increasingly employed for early cancer diagnosis. The critical challenge is finding out precisely what we need to look at before opening the "black box" information and developing a plan to predict it. Although research and development are still underway in this field, it has been shown that combining AI and ML for risk analysis, medical decision support, and early warning of the formation of an epidemic is beneficial. The field of medicine is complex and challenging in and of itself. AI technology has demonstrated promising results in automation and efficient data processing across various commercial sectors, including the financial technology industry. The healthcare sector is predicted to undergo a rethink and upheaval thanks to AI. This may entail using ML to create efficient treatment plans and helping experts analyse medical data. AI in medical research is centred mainly on biomedicine, patient data management, and information retrieval techniques in the various medical technology and industry sectors. Various works of literature and databases provide sufficient information regarding how genomic data of plants and animals can be used with AI-based algorithms that comprehend everything in a proper cube [12,51, 62, 63, 64, 65, 66, 67]. Additionally, enough money is budgeted for research and investments to advance augmented intelligence. In this sector, expert medical judgment is combined with the strength of scientific facts. Big names in the pharmaceutical business are increasingly turning to AI and ML approaches to tackle the practical challenge during drug development. Case studies span a wide range of therapeutic areas, including therapies for cancer, immuno-oncology medications, and metabolic illnesses [68,70]. Beyond the conventional long-haul method, AI technologies may accelerate the fundamental processes of early-stage candidate selection and mechanism discovery. AI and ML technologies can also forecast the onset of certain epidemics and track their global distribution. The forecast can be made using historical data that is available online, satellite data, current social media posts, and other sources. SVM and ANN have recently been used to predict the spread of malaria and, subsequently, the favourable cases of infected persons. The prediction approach may forecast the average monthly rainfall in a particular location, the temperature in a specific month, and other factors are additional valuable data points. Support vector machines and artificial neural networks have recently been used to predict the spread of malaria and, subsequently, the favourable cases of infected persons. The prediction approach may forecast the average monthly rainfall in a particular location, the temperature in a specific month, and other factors are additional valuable data points. Next-generation intelligent electronic health records use AI and ML to aid clinical decision-making, diagnosis, and individualised therapy recommendations [51, 71]. This technology adapts well to various institutions and medical circumstances and is versatile in language and training data.

## Challenges of ML in Medical Sciences:

ML is widely used in healthcare to ease the task, reduce the large data and reduce the time consumed in delivering healthcare.With the wide applications of ML, it comes with challenges. Thanks to AI and ML, computer programs may predict outcomes more correctly without being explicitly instructed. By processing healthcare data in novel ways, improving patient care, and reducing administrative procedures, ML has the potential to revolutionise the medical sector. ML projects in the healthcare sector may now leverage input data from medical records previously required to be examined by humans [72]. ML can carry out human-like tasks because of its capacity to adapt to new inputs and learn from experience. ML can greatly improve the healthcare system since it may reduce subjectivity and unpredictability in clinical diagnosis. It has helped physicians diagnose diseases, tumours, rare syndromes, and exciting cancer [73, 74]. Today, it is common to employ AI and ML exaggeratedly. ML can greatly improve the healthcare system since it may reduce subjectivity and unpredictability in clinical diagnosis. It has helped physicians diagnose diseases, tumours, rare syndromes, and exciting cancer [72, 73]. Today, it is common to employ AI and ML exaggeratedly. Given the enormous potential in this subject, this may be the case. The number of AI consulting firms has increased significantly in recent years. The United States Bureau of Labor Statistics estimates a 13% increase in computer-related professions between 2016 and 2026, which Ziprecruiter.com cites as evidence of the bright prognosis for AI careers [74, 75]. Most readers of this article are probably already familiar with ML and the pertinent algorithms used to categorise or predict outcomes based on data. ML is not the solution to every issue and must be understood. Given ML's value, it can be challenging to acknowledge when it is not the best way to solve a particular issue. It is undeniable that the medical AI sector is rife with cutting-edge developments that appear at an incredible rate. It is simple to assume that medical facilities will eventually use AI to replace doctors. However, one of the essential components of high-quality healthcare is empathy. It enhances patient happiness and encourages recovery. Although AI can perform various activities better than doctors, it cannot replace human beings. Unfortunately, the main defence used against autonomous AI in healthcare is that machines cannot exhibit empathy. A real-life medical professional is the only one who can guide a patient through a challenging treatment process, hold their hand when they get life-altering diagnostic news, occupy a young patient who is afraid of getting blood, or really care about their patients. Real-world examples are used to train ML algorithms. They perform better when more data is provided to them [76, 77]. A closer examination of AI solutions reveals that they operate most effectively in stable and predictable environments. Gigabytes of data can be combed through to find trends and hidden irregularities. However, when a series of specific actions are required, what about complicated activities? Experts claim that for non-linear activities, we are still a long way from being able to replace human medical personnel with AI completely. The same is true for sophisticated treatment procedures. Human monitoring is still necessary despite the advancements made by AI in medicine. For instance, surgical robots operate rationally rather than compassionately. Health experts may recognise important behavioural indicators to help identify or prevent medical disorders. To be efficiently used, AI needs human input and assessment. As AI advances, the tech and medical industries collaborate to further the technology. Frequently, a patient's needs transcend beyond their present physical conditions. The right course of action for a certain patient might vary depending on their social, economic, and historical conditions [28, 42,55,57]. For instance, an AI system could be able to place a patient in a certain care facility based on a specific diagnosis. However, this method may not consider the patient's budgetary constraints or other particular preferences. Medical AI mainly depends on diagnostic knowledge acquired from millions of recorded incidents. A mistake is possible when there is insufficient knowledge about specific diseases, demography, or environmental factors [10]. When recommending a specific treatment, this factor becomes extremely crucial. AI systems are vulnerable to security threats since AI is typically dependent on data networks. If our information is attacked, the expected result might get manipulated, and the desired result will be impacted, which can prove fatal for serious cases. So, using our current knowledge, we may conclude that AI benefits healthcare systems. By automating time-consuming tasks, physicians may free up more time in their schedules to spend with patients. Increasing data accessibility aids healthcare professionals in taking the proper steps to prevent sickness [42]. AI is used to cut administrative mistakes and save crucial resources. Real-time data may be used to inform diagnoses more effectively and promptly. Although there are still challenges and restrictions, the application of AI in healthcare is expanding. AI still needs human supervision. Despite AI's challenges and limitations, the medical sector has much to gain from this revolutionary technology. AI is improving everyone's lives, whether they are patients or medical professionals. In ML, an area of AI, the system is taught to learn from data. ML uses an algorithm that helps the software application predict outcomes without explicitly programming [28, 42, 55,57]. The algorithms utilise data to predict outcomes based on patterns. During prediction, the algorithm is trained by feeding a dataset and can accurately guess the outcome when provided with a new dataset. The prediction has various applications in healthcare or medical science, such as clinical decision support systems. The efficiency and effectiveness of medical data analysis increase immensely by applying ML techniques in the healthcare sector.

## Diverseness of Data:

The data currently present in the healthcare system is heterogeneous. When ML algorithms are applied in healthcare, the learning is solely based on observational data, which leads to many challenges in building models that can answer random questions [78]. While creating the dataset for healthcare, some of the data may be missing, some data may be fragmented, and some data may be duplicated in the dataset, so the machine learns from the incomplete or missing data, because of which the prediction done by the ML algorithms may be misleading. So, to integrate ML models in healthcare, we must create efficient data management policies across all tiers. In ML, for most algorithms, it is assumed that all the features are independent, but in the healthcare dataset, one feature might be dependent on the other, which may lead to irrelevance in the result [78, 79].

## Scarcity of Qualified Resources:

The shortage of Data Scientists and ML Engineers is also one of the top challenges in using ML in healthcare. Many humans and computational resources are required, and it takes time to train the human resources up to the level they can train the machine error-free in the proper direction after annotating the complete data [80].

## Bias in ML Models:

When an ML task is being performed, there can be partiality in the data. For example, there can be more data about a specific class and less data about another class, so the model developed by ML would achieve more accuracy for the class with more data, while it would not perform well on the class with fewer data [81].

## Data governance and privacy: 

Medical information remains private and is not accessible. However, only 17% of respondents in a UK Well come Foundation poll said they opposed disclosing their medical information to other parties [82].

## Open-source algorithms:

Transparent algorithms are necessary not simply to comply with stringent drug development standards but also so that people can comprehend how precisely algorithms create findings [83].

## Optimising electronic records:

Much-fragmented information across several databases requires more outstanding organization due to the ever-increasing volume of data. Personal therapy options will progress as soon as this issue improves [84].

## Recognising the value of data silos:

The healthcare sector must alter its perception of the value of data and its potential long-term benefits. For instance, pharmaceutical firms often hesitate to alter their product strategy or research efforts without apparent financial gains [85].

## Data quality:

ML is based on the utilisation of data to make accurate predictions. Hence, data quality is one of the main parameters that can change prediction results. If there is a presence of incomplete, unstructured, unlabelled, or biased data, the quality of the data gets affected, resulting in more chances of error in prediction or even false prediction, which can impact its application in medical science and the healthcare sector immensely [86]. For using ML in medical science, we need to ensure that our data's quality is good to avoid any wrong prediction changes [87, 88].

## Data quantity:

In addition to data quality, the quantity of data also plays a significant role in accurate prediction; for example, if we are using any ML model, we need to provide it with enough training data to produce reliable results, and if we do not provide enough data, the prediction accuracy of the model gets reduced [86, 89].

## Complexity:

In most ML models, the determination is based on one-to-one association. In this model, we only train our dataset to predict an outcome based on a specific factor. Multiple medical factors might come into play when predicting an outcome or diagnosing a condition using an algorithm. An ML model can predict an inaccurate outcome with a low confidence score in that condition. Hence, the chances of the wrong prediction increase when an ML algorithm deals with complex data. As the data's complexity increases, prediction error also increases [33].

## Manipulation:

In some cases, algorithms may make incorrect predictions because of the manipulation of the model, such as the addition of noise or rotation. In addition, some intentional manipulation can be performed on the model to give the wrong prediction in favour of a specific outcome [33].

## Algorithmic bias:

Algorithmic bias refers to the unintentional generation of an "unfair outcome" favouring one category or condition over another. This problem arises from the generalisation of the training dataset, which leads to inaccurate predictions [81]. For example, when used among other patients, an algorithm aimed to classify benign and malignant moles, trained with the dataset having the images of skin lesions for fair skin patients, can show false predictions. Algorithmic bias can be divided into three components:

• Model bias refers to the prediction model's biases favouring the majority group.

• Variance refers to the model's change due to the model's training with incomplete or missing data from one group.

• Noise- refers to the variable introduced in the model that affects the model's prediction accuracy.

## Conclusion:

It can be concluded that though there can be a wide range of application of ML in healthcare, and it might prove to be a game-changer for the healthcare system, there are many challenges which might pose hindrance which needs to be overcome for the best results. ML in healthcare technology directly impacts the future of advanced medical diagnostics and treatment that uses ML, which has strong analytical capabilities. The future of AI in health care may involve tasks ranging from simple to complex, including answering the phone, reviewing medical records, population health trending and analytics, therapeutic drug and device design, reading radiology images, developing clinical diagnoses and treatment plans, and even conversing with patients. AI can assist with many of healthcare's major problems, but we are still far from making this a reality. Making this a reality is hampered by the problem of data. No matter how cutting-edge the technology and ML algorithms have grown, we cannot wholly use AI in healthcare without enough and well-represented data. The healthcare industry has to digitise patient information, settle on standardised data architecture, and create a flawless system for managing patient data and protecting patient confidentiality. Without these significant alliances and changes in the healthcare industry, it would be challenging to realise AI's full potential for assistance. The powerful capabilities of ML for sorting and categorising health data and expediting doctors' clinical decisions and any kind of predictions that can save lives or make surgery less complicated get the highest grade for the importance of ML's advantages in healthcare. Healthcare informatics uses ML, which has strong analytical capabilities. Consequently, the electronic information given to clinicians is improving significantly. Physicians then put together a treatment plan to support patients and provide the finest care possible. The cost of the surgery may be estimated with the aid of potential results, making therapy more accessible. Although there have been a lot of developments and advances with ML in healthcare, there are various gaps that must be improved before going with full-proof AI in medical sciences.

## Ethical Responsibilities:

This study does not directly involve humans or other organisms as it is a review article.

## Consent for publication:

All authors consent to submit the manuscript to the journal.

## Availability of data and material:

No supplementary material is available.
